# Infected with Scabies Again? Focus in Management in Long-Term Care Facilities

**DOI:** 10.3390/diseases7010003

**Published:** 2018-12-29

**Authors:** Chong Yau Ong, Farhad Fakhrudin Vasanwala

**Affiliations:** 1 Department of Family Medicine, Division of Medicine, Sengkang General Hospital, Singapore 544886, Singapore; farhad.fakhrudin.vasanwala@singhealth.com.sg; 2 SingHealth Duke-NUS Family Medicine Academic Clinical Programme, Singapore 544886, Singapore

**Keywords:** scabies, mites, long-term care

## Abstract

Scabies is a significant public health condition in long-term care facilities, plaguing even developed countries. Although treatments are available, eradication and control of scabies cases still remain a challenge due to delays in diagnosis and difficulties in maintaining preventive and surveillance measures. Prompt treatment of patients and their contacts that are affected, along with concomitant education of health staff and family members, are paramount. Environmental disinfestation is also a concern.

## 1. Introduction

Scabies is often a neglected parasitic disease. It has long been known to human beings, first described by the renowned physician Jeremy Thriverius of the Habsburgian Low Countries during the 16th century. The causal relationship between skin infestation and the scabies mite was first established by Giovan Cosimo Bonomo, an Italian physician, and the apothecary Diacinto Cestoni [1]. Scabies is a significant public health condition both in resource-poor and developed countries [2], affecting individuals of every age and socioeconomic status [3]. Incidentally, the role of poor hygiene in scabies occurrence has been overestimated and is probably more attributable to overcrowding [4,5,6]; this is noted in institutional outbreaks, where high standards of cleanliness are observed [7,8]. Outbreaks in residential and long-term care facilities, however, are usually caused by diagnosis delay and are therefore difficult to control [9].

### 1.1. Epidemiology

A systematic review of population-based studies found the highest prevalence of scabies in Papua New Guinea, Panama, and Fiji [10,11]. Scabies caused 0.21% of Disability-Adjusted Life Years (DALYs) from all conditions studied by the Global Burden of Disease (GBD) 2015 [11]. A review of institutional scabies outbreaks globally revealed that 48% of the outbreaks occurred in residential care facilities for the elderly [12,13]. Prevalence of institutional scabies is probably underestimated [14].

In one review of 206 outbreaks in elderly care facilities caused by 37 pathogens, scabies was the fifth most reported pathogen after influenza and noroviruses, *Salmonella* spp., and Group A *Streptococcus* [15]. Scabies has high median attack rates for health care workers at 36%, only slightly less than *Chlamydia pneumonia* (41%) and noroviruses (42%) [15].

### 1.2. Transmission

This ectoparasite infestation is caused by the mite *Sarcoptes scabiei* variety *hominis*. *S. scabiei* is a member of the family Sarcoptidae, within the class Arachnida. Both male and female mites are invisible to the unaided eye; the maximum adult size is 0.45 mm [4,16].

Scabies is transmitted through skin-to-skin contact, though less frequently through fomites (inanimate objects capable of transmitting an infectious organism such as clothing, towels, and bed linens) [17,18,19,20]. Among adults, sexual contact is an established mode of transmission [3].

Mites dislodged from an infested individual use odor and heat to locate a new host [4]. The probability of being infected is related to the number of mites on the infested individual and the length of contact [3,17].

Away from the host, mites are able to survive and stay capable of infestation for 24–36 h at 21 °C with 40–80% relative humidity [21]. In colder temperatures and higher humidity, they can survive even longer. The mites have been reported to be capable of survival for 19 days at 10 °C and 97% relative humidity, although they are unable to move and penetrate skin at temperatures below 20 °C [4,21]. Scabies mites survive less than 24 h in a temperature of 34 °C [2]. To a lesser extent, transmission can happen through fomites [18,19,21,22,23].

### 1.3. Parasite Lifecycle and Incubation Period

Female mites burrow into the epidermis, while male mites explore the skin for an unfertilized female. Female mites live for 4–6 weeks, producing 2–4 ova a day [16,24]. A single female mite can produce up to 40 ova during her lifetime, the larvae hatching 2-4 days thereafter. Larvae molt into protonymphs (3–4 days) and then tritonymphs (2–5 days) before turning into adult male or female mites (5–6 days). In total, mature adults develop within 10 to 14 days [2].

The incubation period for naïve patients without previous exposure to scabies is 2–6 weeks. However, this period is shorter in people who have been previously infested, whereby symptoms typically develop within 1 to 5 days of re-exposure due to rapid sensitization [3,18].

## 2. Clinical Presentation

The two major clinical variants of scabies are classic and crusted. Classic scabies, the most common presentation, is associated with a relatively low mite burden (approximately 10–15 mites on the body). Crusted scabies usually occurs in older adults, individuals with dementia, immunocompromised individuals, and individuals with severe neurological disease [9,25,26]. It is associated with a higher mite burden of up to millions of mites on the body [27]. Other forms of scabies include bullous scabies that can mimic bullous pemphigoid, scabies incognito, and hidden scabies [28].

### 2.1. Distribution of Rash

The pathognomonic signs of scabies are burrows, erythematous papules, along with the symptom of pruritus (nocturnal predominance) [2,4]. Burrows are serpiginous, whitish lines in the upper epidermis, measuring several millimeters in length. Typical areas where signs of infestation can be observed are the interdigital spaces of the hand, flexural aspect of the wrists, elbows, penis shaft, nipples, buttocks, axillae, and periumbilical area.

In infants and the elderly, classic scabies can present atypically on the head, face, back, and diaper area [2,24,29]. Crusted (or Norwegian) scabies affect patients with HIV-infection, human T-cell lymphotropic virus type 1, other immunocompromised patients, and those with sensory and motor neuropathy or dementia [29,30,31,32]. Sometimes it affects persons without apparent risk factors [3]. Lesions are described as erythematous, hyperkeratotic, psoriasiform, warty, and exfoliating, scaly rash over the scalp, face, fingers, genitalia, and even nails [4,29]. Inappropriate long-term application of potent topical steroids, especially in the elderly, can lead to crusted scabies [29]. 

### 2.2. Host Immune Response

Delayed type IV hypersensitivity, established as a mechanism leading to signs and symptoms of scabies, occurs as a reaction against the scabies mite’s saliva, eggs, or feces (sycbala) [18]. The reaction can be delayed for up to four weeks, which accounts for long latency of the disease [33].

Both cell-mediated host immune response and humoral response play roles in the host immune response [2,18]. Increased serum levels of IgG and IgE (combined with peripheral eosinophilia) are not protective against reinfestation [2].

### 2.3. Complications

Scabies mites are not known to transmit secondary infections. However, severe scratching can lead to secondary skin infection. Secondary skin infections are not limited to boils, cellulitis, pyoderma, or lymphangitis due to *Streptococcal pyogenes*. Streptococci and staphylococci have been isolated from skin burrows as well as mite fecal pellets, suggesting that the mites themselves may contribute to the spread of pathogenic bacteria [5]. Bacterial superinfections, however, are uncommon in immunocompetent adults living in Western countries [34]. Secondary infection of scabies with *S. pyogenes* is a major precipitant of acute post streptococcal glomerulonephritis and possibly rheumatic fever [4,35].

## 3. Diagnosis

Diagnosis is based on the contact history of the patients, health care workers, or even family members. The combination of pruritic eruptions, characteristic lesions and their distribution, and the identification of mites, eggs, or feces on skin scrapings confirm the diagnosis.

In practice, burrows are often obliterated by bathing, scratching, formation of crusts, or superinfection [4]. Visibility of burrows can be improved with an ink burrow test, where burrows will absorb the ink and be readily apparent as ink-filled wavy lines where the mite has tunneled, called the stratum corneum [36]. 

The usual method of obtaining skin samples is accomplished through skin scraping. In this method, the scalpel should ideally be oil-covered as the oil helps to keep the scraped content adhering to the blade [2]. Multiple superficial skin samples should be obtained from characteristic lesions by scraping laterally across the skin cautiously to avoid bleeding [24]. Scrapings are then placed on a covered slide for direct microscopic examination. 

Video dermatoscopy is suitable for clinching diagnosis in children. With a magnification of up to 600 times, mites and burrows can be identified [37]. Use of a handheld dermatoscope requires training to recognize the typical “jet with condensation trail” pattern. Performed by a trained practitioner, dermatoscopy yields high accuracy in diagnosing scabies [38,39].

Epiluminescence microscopy using dermatoscopy has also been used in dermatology clinics to identify in vivo mites with good sensitivity [40]. Incident light microscopy (with a magnification of up to 200×) and reflectance-mode confocal microscopy have also been found to have high diagnostic sensitivity [41,42]. Serology tests have yet to be successful in human infestations [43]. Complementary DNA libraries have been constructed for *S scabiei* var. *hominis*, but commercial molecular diagnostic tests have not yet been developed [38,44,45].

The International Alliance for the Control of Scabies (IACS) recently released a consensus on diagnosis of scabies with high agreement (Table 1) [46].

Identification and early treatment of suspected scabies is critical especially in residential or care facilities. Delays in diagnosis have been reported in nursing homes where it was misdiagnosed as eczema and other skin conditions by visiting general practitioners (GPs) until that diagnosis was superseded by another GP [25]. Most nursing homes and institutional residential or care facilities do not have access to specialist dermatological support [25]. 

### Differential Diagnoses

The list of differential diagnoses is extensive and includes atopic dermatitis, contact dermatitis, folliculitis, impetigo, papular urticarial, bites (from midges, fleas, lice, bedbugs, and other mites), and tinea [2,3]. Nearly all pruritic dermatoses have to be considered differential diagnoses [4]. 

## 4. Management

### 4.1. Principles of Treatment

The principle of treatment of scabies is rapid isolation and treatment of the index case, identifying contacts, and environmental disinfestations [47]. It is imperative for the close contacts of individuals diagnosed with scabies to be treated simultaneously because they may have been infected without yet manifesting the symptoms, and so act as reservoirs for infection [48,49,50,51]. Isolation and locking of doors for residents with dementia and wandering behavior is essential, although it can be distressing to them and staff [25].

### 4.2. Topical and Oral Agents

Most scabies infestations are treatable with scabicides. It is essential that steps for environmental disinfection take place simultaneously with medical treatment. Topical treatments typically require application from the neck down to the soles of the feet (including fingernails and toenails) for duration of many hours. There is no international consensus on the appropriate schedule of treatment, and recommendations in one jurisdiction may not be applicable in others [14,48,52]. 

In a review of interventions for scabies, permethrin was found to be more effective than other scabicides [53]. A recent review found no difference detected in the efficacy of permethrin in comparison to ivermectin [54]. Although malathion has been used with success in many centers, there are no trials to compare the effectiveness of malathion against other scabicides [53]. Table 2 summarizes commonly used treatments for scabies. 

Antihistamines and emollients are useful for symptomatic management of itch, including medication-related post-scabetic itch [49]. Topical keratolytics such as salicylic acid can be used to treat crusted scabies. It is applied on days where scabicide is not applied.

### 4.3. Drug Resistance and Other Treatments

Of late, the resistance to scabicides has been increasingly reported [55,56,57,58,59]. Four different players that could potentially contribute to scabicide resistance have been identified as follows: (a) voltage-gated sodium channels, (b) glutathione S-transferase (GST), (c) ATP-binding cassette transporters, and (d) ligand-gated chloride channels [57].

Moxidectin (an established treatment of scabies in dogs and sheep) is currently being evaluated as an oral agent for scabies. It is related to ivermectin and has the same mechanism of action, but is more lipophilic (retains in tissue longer). The prospect of moxidectin as future therapy for scabies has been promising [60,61,62].

Vaccination has been shown to have some potential in controlling scabies epidemics [63]. The development of a vaccine against scabies is still being conducted [57,64]. 

### 4.4. Environmental Disinfestation

Isolation rooms should be cleansed thoroughly. Residential and care facility staff should avoid direct skin-to-skin contact by using protective garments such as gowns and gloves. Correct handling of disposed protective garments should also be observed.

Infested individuals’ bedding, clothing, towels, and personal bed jackets should be separately machine-laundered using hot water above 75 °C, followed by hot dryer cycles. No special processing such as autoclaving or bleaching is required [16]. Items that are unable to be laundered, such as shoes, should be placed in a plastic bag and left for 72 h [16,19]. Amenities and equipment such as geriatric chairs, commodes, and toilets should not be shared until 24 h post treatment [19]. Chloramine 5% has been used to disinfect rooms of infested individuals [47].

### 4.5. Management of Complications or Treatment Failure

Resolution of active lesions and alleviation of pruritus indicate that therapy has likely been successful. Having said that, pruritus may persist two to four weeks after successful treatment and can be part of the resolution process or caused by post-scabetic dermatitis. In this case, scabies scrapes can be repeated post wash-out of topical scabicides to confirm eradication.

Confirmed treatment failure can be largely attributed to ineffective application of topical scabicides and incomplete environmental control [65]. Alternative therapies should be considered in the case of resistance to initial therapies.

### 4.6. Management of Outbreak and Prevention Program

**Figure 1:**
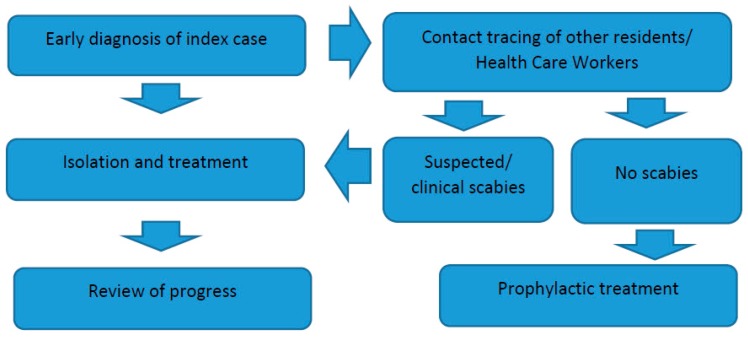
Workflow for management of scabies in long term care facilities.

In residential homes and care facilities, early recognition of scabies is essential to prevent outbreaks [50]. Diagnosis may be delayed because of the less familiar way that scabies can present in the elderly [66]. Once an outbreak occurs, prompt control of the index patient and rapid tracing of contacts to identify secondary cases are necessary (Figure 1) [48,49]. When prolonged exposure to a case of scabies results in multiple secondary cases, institution of simultaneous mass prophylaxis is the most efficient strategy for terminating the outbreak and can be implemented without ward closure, although the logistic aspects are considerable [50]. However, an aggressive approach of the minimization of transmission pathways such as the reduction of staff rotation, cancellation of community activities, and if possible, of new admissions (ward closure), has also been recommended [67].

Due to the unavailability of well-designed, randomized controlled trials (RCTs) to provide conclusive evidence of prophylactic measures, it is unclear whether prophylaxis is more appropriate than a “wait and see” approach, whereby contacts are educated regarding the possibility of infection and advised to seek medical consultation should they develop symptoms suggestive of infection [48,49].

In the review of prevention strategies, the authors summarized the concerns and barriers for prophylaxis (Table 3) [48]. In general, control of large outbreaks is distressing and requires significant effort in terms of time, money, organization, coordination, and teamwork among healthcare staff [25,68].

Long-term care facilities should possess a scabies prevention program. Such a program should include assessment of skin, hair, and nails for all new admissions as they arrive [72]. Any lesions suspected to be scabies and other dermatological conditions should be escalated to the physician. Time should be allocated to conduct this assessment periodically. However, in practice, long-term care facilities often suffer inadequate staffing.

Prolonged surveillance in the eradication of scabies in long-term care facilities is needed due to the dynamics of residents and rotation of staff [73]. New cases can occur due to the transfer of new residents with unrecognized scabies, as well as existing residents who returned to the facilities after contracting scabies from another hospital.

### 4.7. Contact Tracing

Concurrent treatment of contacts and individuals diagnosed with scabies is important, as the onset of symptoms is often delayed and therefore contacts may have active scabies while they are asymptomatic of pruritus. Family members that co-habit, including domestic workers, as well as family members and visitors of the diagnosed individuals up to six weeks prior to the diagnosis should be identified and treated.

In residential and care facilities, all persons that are in contact with the affected individuals should be traced and treated. This includes doctors, nurses, social workers, volunteers, therapists, assistants, porters, security officers, and visitors. This can be done through the checking of electronic systems used for keeping track of staff and visitors who enter the affected ward or cubicle. Registration of staff or visitors using books should be done and subsequently traced during an outbreak if the entrances to the wards are not digitally captured. This can be logistically challenging in nursing homes and care facilities with day care services, including short and long term patients [47]. Identified contacts should be treated with the same regimen used for classic scabies. Cooperation and compliance of health care workers and visitors is needed for successful treatment [74].

Restriction of staff rotation in the care facility has been identified as one of the steps of the successful control of outbreaks [73]. Nurses who are caring for symptomatic patients and residents in the same ward are required to examine themselves regularly, and if found to be symptomatic, are instructed to contact the ward employee in charge in order to be excused from work [75]. Infested staff can return to work 24 h after their first scabicide treatment [19].

**Figure 2:**
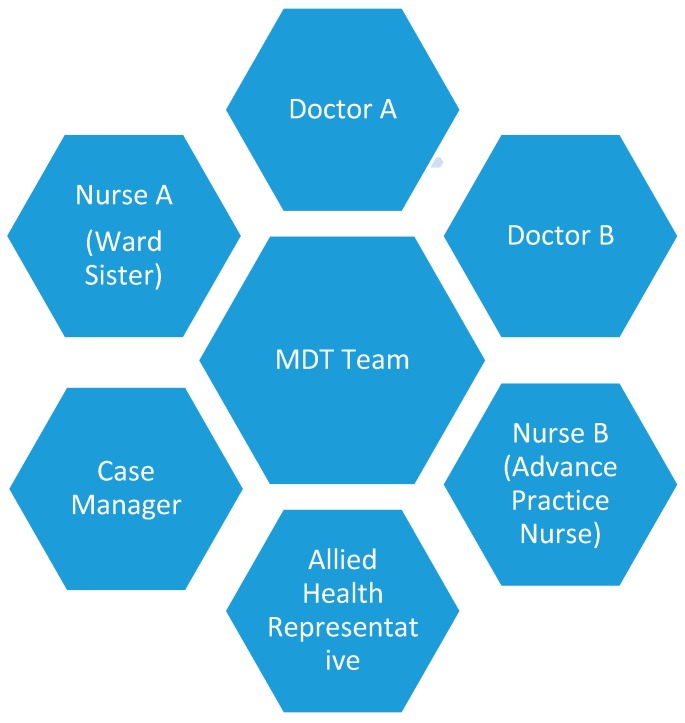
Multi-disciplinary (MDT) Team. Doctor A: Chair the discussion and oversee the control steps. Reports to higher management on the care plans. Nurse A: Oversees the screening of nurses and prophylactic treatments. Ensures the nurses adhere to contact precautions and hand hygiene. Supervises the cleaning of fomites and environment. Case Manager: Trace all contacts of the diagnosed individual through digital or paper records. Contact them and refer them for treatment. Doctor B and Nurse B: Treat and reassess all cases of scabies. Provide treatment for contacts. Allied Health Representative: Ensure the allied health and other health care workers adhere to contact precautions and hand hygiene. Assist Case Manager in contact tracing.

In resource-adequate settings, we suggest a multi-disciplinary team consisting of a minimum of six members to address the treatment and preventive aspects of scabies infestation (Figure 2). Meetings should be conducted to update the progress of the treatment of cases and their contacts and identification of new cases [67]. In resource-limited settings, public health nurses or trained individuals can be tasked to chair, coordinating the preventive measures.

## 5. Recommendations

It is imperative that early identification of the index case and subsequent contact tracing be performed during the onset of an outbreak; followed by treatment are the principles of management. High adherence to contact precautions and fomite cleaning are important steps that are often ignored. 

Finally, continuous, vigilant surveillance of scabies in residential and long-term care facilities and activation of dedicated MDT teams to address the outbreak is the cornerstone of curbing scabies infestation in a long-term care institution.

## Figures and Tables

**Table 1:** The 2018 International Alliance for the Control of Scabies (IACS) criteria for the diagnosis of scabies.

A: Confirmed scabies At least one of: A1: Mites, eggs, or feces on light microscopy of skin samples A2: Mites, eggs, or feces visualized on individual using high-powered imaging device A3: Mite visualized on individual using dermoscopy B: Clinical scabies At least one of: B1: Scabies burrows B2: Typical lesions affecting male genitalia B3: Typical lesions in a typical distribution and two history features C: Suspected scabies One of: C1: Typical lesions in a typical distribution and one history feature C2: Atypical lesions or atypical distribution and two history features History features H1: Itch H2: Close contact with an individual who has itch or typical lesions in a typical distribution *Notes*: 1. *These criteria should be used in conjunction with the full explanatory notes and definitions (in preparation)*. 2. *Diagnosis can be made at one of the three levels (A, B, or C)*. 3. *A diagnosis of clinical and suspected scabies should only be made if other differential diagnoses are considered less likely than scabies*.

**Table 2:** Summary of treatments for scabies.

Drug Name and Preparation	Dosage and Instruction	Major Side Effects	Notes or Contraindications
**Topical**			
Permethrin	5% cream. Rinse off after 8–14 h. Second application one week after the first. Crusted scabies: Apply daily for 7 days, then 2x/week until cured. Combination therapy with oral ivermectin.	Itch and sting on application.	Not to be used in infants under age two months. Can be used in infants and breastfeeding mothers.
Benzyl benzoate	10–25% lotion. Rinse off after 24 h. Alternatively, apply overnight for 2 consecutive days. Second application 1 week after the first. Crusted scabies: Apply daily for 7 days, then 2x/week until cured. Combination therapy with oral ivermectin.	Burning and sting on application.	Not recommended in infants below 6 months (dilutional doses) required. Disulfiram-like effects if alcohol is consumed less than 48 h prior to application.
Crotamiton	10% cream. Apply to nodules for 24 h, rinse off, and reapply for another 24 h.	None	Safety in children has not been established.
Precipitated sulfur	3–6% lotion, 5–40% petrolatum. Apply for 24 h and then reapply every 24 h for the next 2 days. Alternatively, apply overnight for 3 consecutive days.	None	Inexpensive. Used in neonates, pregnant women, and breastfeeding mothers.
Malathion	0.5% lotion. Rinse off after 24 h. Repeat application after one week.	Burning and sting on application.	
**Oral**			
Ivermectin	3 mg tablets. Single dose of 200 mcg/kg body weight. Second dose 2 weeks later. Crusted scabies: 200 mcg/kg/dose on days 1, 2, 8, 9, and 15. Combination use with permethrin/or benzyl benzoate. Apply for 7 days, then 2x/week until cure.		Contraindicated in children less than 15 kg or pregnant and breastfeeding mothers. Absorption can be improved with fatty meals. Care must be taken when administered with drugs that can augment GABA activity (valproate, barbiturates, and benzodiazepines).

Efficacy of one application in comparison to two applications has not been formally tested [16]. Application of topical therapy above the neck level should be considered in children and the elderly who have significant scalp involvement.

**Table 3:** Concerns and challenges in mass prophylaxis.

1) Commitment and willingness of exposed contacts to undergo treatment [69]. 2) Substantiating the degree of contact of exposed individuals with the index case, including those who are unable to consent for treatment [70]. 3) Side effects of therapy [14]. 4) Possibility of drug resistance against anti-scabetic treatments [49,56]. 5) Stigma associated with diagnosis, which may lead to reluctance in disclosure of diagnosis to close contacts [4,12,67]. 6) Cost associated with providing medical treatment to all contacts [12,25,71] and getting medications in bulk [25]. 7) Logistical difficulties in identifying all contacts of an index case [12,25,69].
